# Reference frames for reaching when decoupling eye and target position in depth and direction

**DOI:** 10.1038/srep21646

**Published:** 2016-02-15

**Authors:** A. Bosco, R. Breveglieri, K. Hadjidimitrakis, C. Galletti, P. Fattori

**Affiliations:** 1 Department of Pharmacy and Biotechnology, University of Bologna, Bologna, Italy; 2 Department of Physiology, Monash University, Clayton, Melbourne, Australia

## Abstract

Spatial representations in cortical areas involved in reaching movements were traditionally studied in a frontoparallel plane where the two-dimensional target location and the movement direction were the only variables to consider in neural computations. No studies so far have characterized the reference frames for reaching considering both depth and directional signals. Here we recorded from single neurons of the medial posterior parietal area V6A during a reaching task where fixation point and reaching targets were decoupled in direction and depth. We found a prevalent mixed encoding of target position, with eye-centered and spatiotopic representations differently balanced in the same neuron. Depth was stronger in defining the reference frame of eye-centered cells, while direction was stronger in defining that of spatiotopic cells. The predominant presence of various typologies of mixed encoding suggests that depth and direction signals are processed on the basis of flexible coordinate systems to ensure optimal motor response.

The reaching movement can be thought of as a vector that starts at the current location of the hand and ends at the reaching target^1^. Information about the location of the reaching target, initially represented in retinotopic coordinates, has to be converted into “a motor” frame of reference. The posterior parietal cortex (PPC) plays an important role in these visuomotor transformations. Many studies have demonstrated the presence of parietal neurons encoding reaching targets in an eye-centered frame of reference^2^,^3^ as well as neurons encoding targets in a spatial and mixed eye- and hand-centered frame of reference^3^,^4^,^5^,^6^,^7^,^8^,^9^. The common limitation of these studies was that they considered only a two-dimensional arrangement of eye/hand positions and did not take into account the depth. However, natural reaching movements are performed in a three-dimensional space (the peripersonal space), where the target’s and the effector’s position can vary in both direction and depth. Two previous studies in monkeys’ PPC investigated the encoding of reaching in depth, but not in direction^10^,^11^. To our knowledge, only one study to date has investigated the reference frames for reaching considering both depth and direction^12^. In that study, carried out in the medial PPC area V6A^13^, the hand started the movement from two different positions to reach for foveated targets, making it possible to distinguish between body-centered (spatial) and hand-centered representations of peripersonal space. The majority of V6A neurons encoded target location either relative to the body or in mixed body- and hand-centered coordinates, whereas ‘pure’ hand-centered representation was present only occasionally. In the Hadjidimitrakis *et al.*^12^ study, the presence of an eye-centered reference frame was not addressed, as reaching targets were always foveated.

The aim of the present study was to investigate the reference frames of reaching targets in V6A by decoupling gaze and target positions in both direction and depth. We trained two monkeys to reach for targets in different eye/target configurations within the peripersonal space, and tested each cell in three task configurations. In the first, reaching targets changed position while fixation targets were kept constant in a central position; in the second, fixation targets varied while reaching targets remained constantly in a central position; in the third, reaching and fixation targets were coincident. By the combination of these three tasks, we were able to analyze the relative contribution of depth and direction signals in target encoding, during both planning and execution phases of reaching.

Our findings support the view that V6A uses mixed types of reference frames where in some cases the encoding is more based on eye/target relative position (unbalanced eye-centered mixed cells) and in others on absolute target position (unbalanced spatiotopic mixed cells). The neural modulation occurred in depth, direction or both, showing complex interaction between the two signals. The presence of a mixed type of reference frame differently balanced among several space representations suggests that a flexible encoding of target position is used in V6A, an encoding process that could ensure optimal motor response within a multisensory environment.

## Results

**Figure 1:**
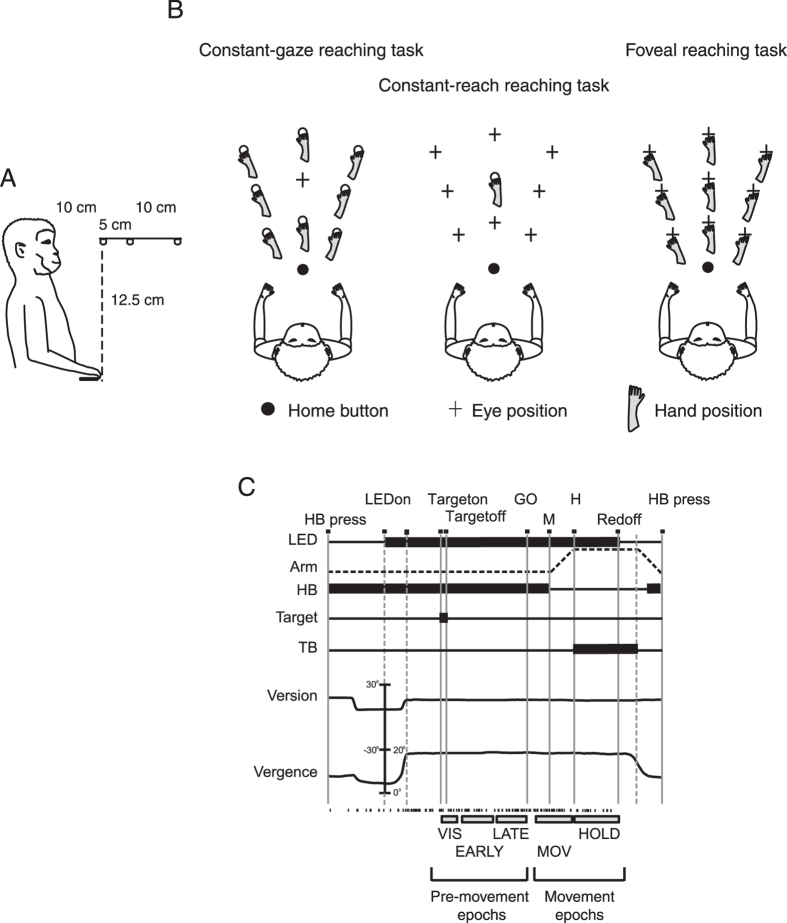
Experimental setup and task sequence. (**A**) Side view of the reaching in depth setup. (**B**) Top view of the three task configurations. Eye and hand movements were performed in darkness toward 1 of the 9 LEDs located at the eye level at different depths and directions with uncoupled (first 2 configurations) and coupled (third configuration) eye (cross) and target position (open circles). Left panel: reaching movements were performed toward 1 of the 9 targets (open circles with hands). The spatial position of the target changed, but gaze was kept constant at the central position, resulting in changes of eye/target configuration. Central panel: reaching movements were performed always toward the target located in the central position of the panel. During the execution of the task, the monkey had to fixate a LED on the panel, which could be in 1 of 9 different positions (cross on the panel), always resulting in different eye/target configurations. Right panel: reaching movements were performed toward 1 of the 9 targets. During the task, the monkey had to fixate and reach for the same target (cross and hand on the panel). The relative position of eye and target remained constant. (**C**) Time sequence of task events with eye target status (LED), arm status, home button (HB) status, Arm target status (Target), target button status (TB), the eye’s vergence and version traces and the spike train of neural activity during a single trial. From left to right, vertical continuous lines indicate: trial start (HB press), eye target appearance (LED on), arm target appearance (Target on), switching off of the arm target (Target off), go signal (GO), start of the arm reaching epoch (M), beginning of the holding of the target (H), switching off of the LED (Red off) and trial end (HB press). Long vertical dashed lines indicate, from left to right, fixation target appearance, the end of the saccade and the start of the return movement, respectively. Bottom rectangles show quantified task epochs: three epochs in the pre-movement period (VIS, EARLY DELAY, LATE DELAY) and two in the hand movement period (MOV, and HOLD).

We used a three-dimensional instructed-delay reaching task with 9 target locations that the monkey had to reach for with different combinations of eye and hand position (Fig. 1), for a total of 27 types of trials. Reaching targets were located at eye level (Fig. 1A), at different depths and in different directions (Fig. 1B). The trial types were grouped in three task configurations (Fig. 1B), identified as ‘Constant-gaze reaching’ where the animal looked at the central target and reached for the peripheral ones, ‘Constant-reach reaching’ where it reached for the central target but looked at all the peripheral ones, and ‘Foveal reaching’ where it reached for the gazed target. This geometry allowed us to describe, first, whether V6A cell firing rates encoded the target position relative to gaze position (eye-centered relative), to target position in space (spatiotopic) or to a combination of these parameters (mixed frame). Second, it enabled us to define the contribution of depth and direction signals to the reference frame employed by the V6A neuronal population. It should be noted that the term “spatiotopic” in these experimental conditions is equivalent to “head-centered” or “body-centered”, as the head was fixed relative to the body, and relative to external space.

We analysed 107 neurons tested for different eye/target configurations. We divided the trial into 5 intervals that describe the time-course of the trial and correspond to pre-movement (VIS, EARLY DELAY and LATE DELAY) and movement-related (MOV and HOLD) phases of the task (Fig. 1C; see Materials and Methods Section, for timing details). For each epoch, we further analysed the cells that presented a significant effect of target position in at least one task (one-way ANOVA; P < 0.05): 99/107 cells were processed for epoch VIS, 105/107 for EARLY DELAY, 102/107 for LATE DELAY, 106/107 for MOV, and 104/107 for HOLD.

**Figure 2:**
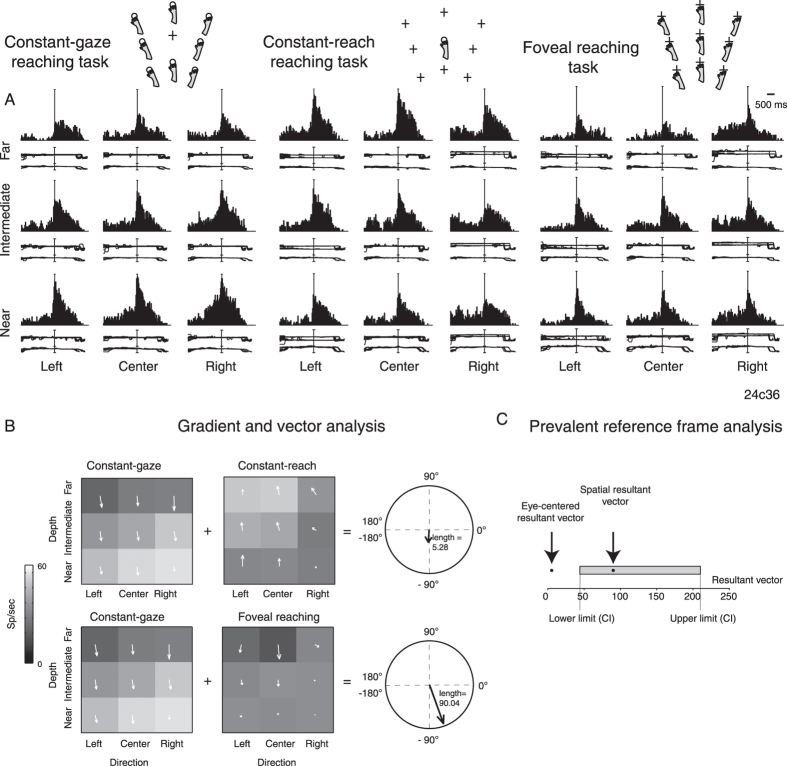
Example of unbalanced eye-centered mixed cell. (**A**) from left to right: spike histograms for Constant-gaze reaching task, Constant-reach reaching task and Foveal reaching task arranged at 3 directions (rows) and 3 depths (columns). The neuron encodes reaching movements in unbalanced eye-centered mixed reference frame showing activation when the animal reached targets nearer than the fixation point in the combination of Constant-gaze reaching and Constant-reach reaching, and when the animal reached the right-near target in the Foveal reaching. Vertical scale: 85 spikes/s. Alignment: movement onset. (**B**) Response fields, vector fields and resultant vector for epoch MOV when reaching movements were made in the combination of Constant-gaze and Constant-reach tasks (eye-centered configuration, top) and in the combination of Constant-gaze and Foveal reaching tasks (spatiotopic configuration, bottom). The 2 pairs of vector fields show the convergence on the peak of activity for targets nearer than the fixated position in the first pair and for targets near to the monkey head in the second pair with the alignment of 2 resultant vectors along the depth dimension (angle: −93.74°, length: 5.28, first pair; angle: −70.19°; length: 90.04, second pair) of polar plot on the right. Upper semicircle ranges from 0° to 180°, Lower semicircle from 0° to −180°. (**C**) Range of normal distribution containing 95% of resampled resultant vectors in the 2 pairs of task configurations considered (CI: [44.11, 218.30]) and corresponding position of real values with respect to CIs.

The majority of cells showed a multiple, complex pattern of activity modulation. The neuron shown in Fig. 2A is an example of the pattern found in area V6A. The cell showed stronger differences in discharges along the depth dimension rather than along direction (compare differences among rows with differences among columns in Fig. 2A), thus suggesting that the cell modulation is mainly extended along the depth dimension. The cell maximally discharged during movement execution for near targets in Constant-gaze configuration and for far targets in Constant-reach configuration. In other words, this cell always showed higher firing when the reaching target was located nearer to the monkey with respect to the fixation position. In Foveal reaching, the cell showed weaker spatial tuning, with a slight preference for near and intermediate targets. The observation of cell activity in the 3 tasks indicates a mixed encoding of reaching target based on the relative position between eye and target and on target position.

**Figure 3:**
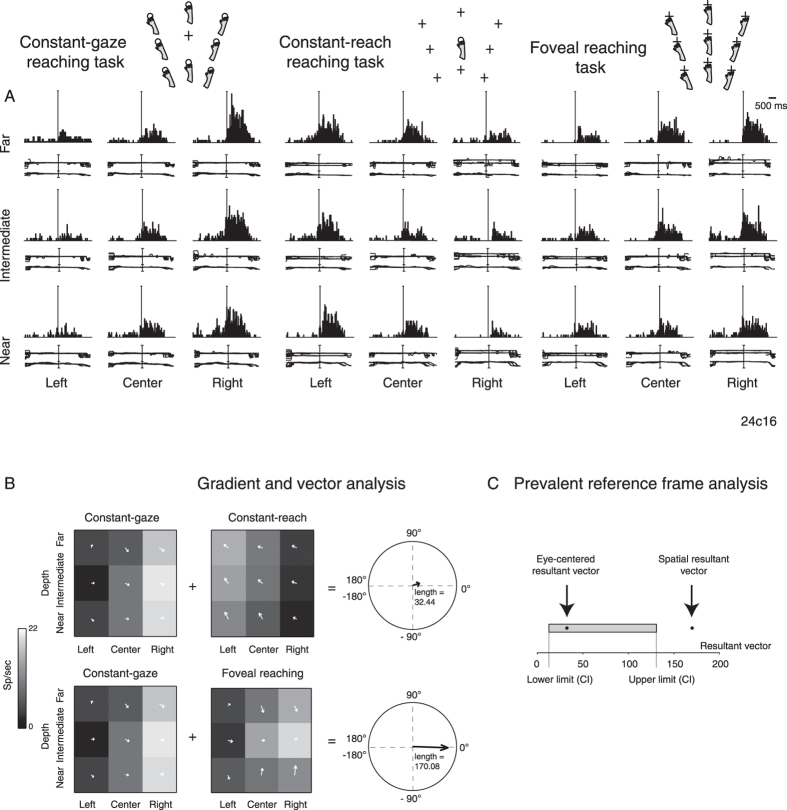
Example of unbalanced spatiotopic mixed cell. (**A**) The neuron encodes the reaching movement in mixed reference frame, unbalanced toward the spatiotopic reference frame. It shows activation when the animal reached toward targets at the right of eye position in the combination of Constant-gaze reaching and Constant-reach reaching, and shows activations when the animal reached the targets located in the right part of the monkey’s working space in Foveal reaching. Vertical scale: 55 spikes/s. Alignment: movement onset. (**B**) The 2 pairs of vector fields, calculated for the epoch MOV, show the convergence on the peak of activity for targets to the right of the eye position in the first pair and for right targets in the second pair with the alignment of 2 resultant vectors along the direction dimension as the polar plots display (angle: −17.39°, length: 32.44, first pair; angle: −2.00°; length: 170.08, second pair). (**C**) Range of normal distribution containing 95% of resampled resultant vectors in the 2 pairs of task configurations considered (CI: [12.70, 130.95]) and corresponding position of real values with respect to CIs. All details as in Fig. 2.

To define the predominant reference frame employed by each cell and their overall spatial preference, we used a combination of gradient and vector analysis which has been used by other Authors to describe the influence of more than one variable simultaneously^14^,^15^,^16^,^64^. Fig. 2B shows an example of this analysis applied to the cell shown in Fig. 2A. Three 3 × 3 gradient fields representing the firing rates for every configuration of eye and reaching target position in each of the three tasks are reported. Each element within the matrices therefore represents the gradient plotted as two-dimensional vector fields. We calculated the length of resultant vector as the sum of the x and y components of each arrow forming the vector field pair (pairs: Constant-gaze reaching/Constant-reach reaching; Constant-gaze reaching/Foveal reaching). The sum of two vector fields (gradient analysis) characterized by opposite directions as in Fig. 2B (top) tends to a null value and, on the contrary, two vector fields with same directional alignment (Fig. 2B, bottom) tend to add together. We statistically determined the prevalent reference frame employed by the cell resampling the resultant vector length in order to obtain lower and upper confidence intervals (CI) that included 95% of values for each pair of configurations analysed. We called the resultant vector resulting from the computation derived from Constant-gaze and Constant-reach configurations as an *eye-centered resultant vector,* and the vector resulting from Constant-gaze and Foveal reach configurations as a *spatial resultant vector*. As the eye-centered representation tends to have opposite directions of vector fields in Constant-gaze and Constant-reach configurations, we defined cells as unbalanced eye-centered mixed cells when the resultant vector was smaller than the lower confidence interval (CI) for this pair of configurations (Fig. 2C), and as unbalanced spatiotopic mixed cells when the resultant vector was larger than the upper CI extracted from the vector fields of Constant-gaze and Foveal reach (Fig. 3C), as the vector fields in these configurations present the same direction. All detailed criteria used to compute the CIs of resultant vectors are defined in Materials and Methods.

The vector fields of Constant-gaze and Constant-reach configurations in Fig. 2B (top) representing the cell in Fig. 2A showed a predominant vertical alignment, but an opposite direction of vectors. The computation of the resultant vector among the two task configurations is presented in the polar plot on the right. It resulted in a general preference for near targets with an angle of −93.74° and a length of 5.28 (Materials and Methods for details). When we compared the Constant-gaze and Foveal task configurations (Fig. 2B, bottom), the two vector fields were perfectly aligned with the same direction of vectors. The resultant vector in the polar plot on the right computed between these two configurations showed an overall preference for near targets defined by an angle of −70.19° and a length of 90.04. In the case of the neuron in Fig. 2A, the eye-centered resultant vector (5.28, see Fig. 2C) was inferior to the lower CI and the spatial resultant (90.04) was included between the two CIs (95% CI [44.11, 218.30]), as reported in Fig. 2C. This quantification defines that the neuron is mixed but, albeit containing representation based on spatial target position as well, predominantly encodes the reaching target based on the *relative position* of the eye and the target.

The discharge of another V6A cell is shown in Fig. 3. The cell is clearly spatially modulated during the execution of arm movement and target holding in all three task arrangements (Fig. 3A). The discharge in Constant-gaze and Constant-reach tasks clearly indicates that this cell was modulated by the relative position between the eye and the reaching target. In Constant-gaze reaching tasks, the preferred positions are located to the right of the eye position. In Constant-reach, the pattern of modulation again showed the highest activity when the target was to the right of the eyes, or, put another way, when the hand reached for targets to the right of the fixation point. In Foveal reaching, where the eye/target relative position remained constant, the best response was for right target positions, suggesting that the eye/target relative position was not the only factor driving neural discharges. Gradient analysis for this cell is depicted in Fig. 3B. It shows that the directional tuning of the matrices and the resultant vectors obtained from the sum of the two pairs of task configurations reveal a general preference for right space (17.39°, eye-centered resultant vector; −2.00° spatial resultant vector). The qualitative analysis suggested that this neuron could encode the position of the reaching target on the basis of eye/target relative position and of target position in space. As for the cell shown in Fig. 2, we quantified the pattern of discharge by the CI computation of 2 vector lengths and found that the space-based coordinate system prevailed on the eye-centered system, as illustrated in Fig. 3C. For this neuron, the resultant vector for eye-centered encoding was 32.44 whereas it was 170.08 for spatial resultant vector (95% CI [12.70, 130.95]). Data analysis indicates that the neuron of Fig. 3, like that of Fig. 2, contained two target representations, but in that of Fig. 3, in contrast to the neuron of Fig. 2, the weight of spatiotopic representation was greater than that of eye-centered representation. In other words, neurons like those of Figs 2 and 3 showed a mixed frame of reference with single frame representations differently balanced in each neuron. We defined these types of neurons “unbalanced mixed cells” because, although they presented both eye-centered and spatiotopic representations, one prevailed over the other.

**Figure 4:**
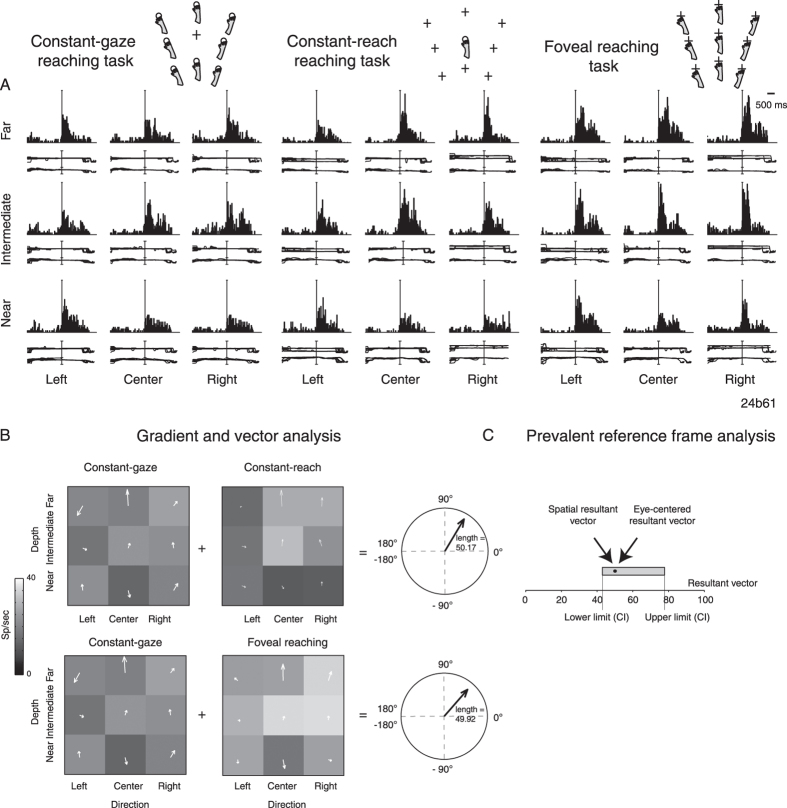
Example of balanced mixed neuron. (**A**) The neuron encodes reaching movements in mixed coordinates, firing during the execution of movement with similar intensity in the 3 task configurations tested. Vertical scale: 75 spikes/s. Alignment: movement onset. (**B**) The 2 pairs of vector fields, calculated for MOV, show similar distribution of vector fields (angle: −59.18°, length: 50.17, first pair; angle: −48.32°; length: 49.92, second pair) and resultant vectors oriented toward the right upper corner, indicating a spatial tuning evoked by both depth and direction. (**C**) Range of normal distribution containing 95% of resampled resultant vectors in the 2 pairs of task configurations considered (CI: [42.99, 77.71]) and corresponding position of real values with respect to CIs. All details as in Fig. 2.

Other cells employing a mixed frame of reference showed more balanced representation and were defined as “balanced mixed cells”. One of these is presented in Fig. 4A. The neuron showed a clear activity during the execution of arm movement and target holding, with a similar scheme of modulation in the three task configurations. This similar trend in the 3 tasks is captured by the vector fields in Fig. 4B. The general spatial trend of the two resultant vectors pointed to the upper right corner with angles of 59.18° for eye-centered resultant and 48.32° for spatial resultant vectors, respectively. This indicates a spatial tuning of reaching activity along both direction and depth dimensions. As Fig. 4C shows, the resultant vectors displayed comparable lengths that corresponded to 50.17 for eye-centered resultant vectors and 49.92 for spatial resultant vectors (95% CI [42.99, 77.71]). The two values of vector were contained within CIs suggesting a balanced mixed encoding based on eye/target relative position or/and on absolute target position with comparable weight and developed along depth and direction dimensions.

**Figure 5:**
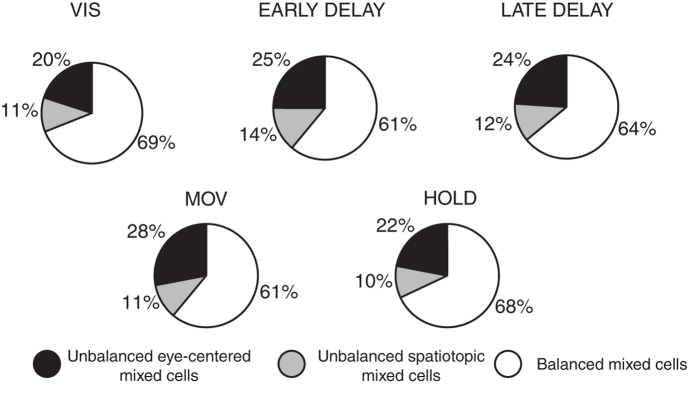
Reference frame quantification in V6A population. Incidence, epoch per epoch, of the percentage of neurons presenting unbalanced mixed eye-centered (black), unbalanced mixed spatial (grey) and balanced mixed (white) encoding of target position.

The 3 typologies in which reference frames are expressed in V6A (like the 3 examples in Figs 2, 3, 4) represented a single mixed encoding where eye-centered and spatiotopic representations present different weights. The relative incidence of these 3 typologies is shown in Fig. 5, where, for each epoch analysed, we show the percentage of cells using the different variations of mixed frames of reference. For all task intervals, the predominant model of coordinate system was the balanced mixed cells where the percentages ranged from 59% to 69% in all epochs (specifically, 69% in VIS; 61% in EARLY DELAY; 64% in LATE DELAY, 61% in MOV, 68% in HOLD). Unbalanced eye-centered mixed encoding was present in the population between 20% and 28% (20% VIS; 25% EARLY DELAY; 24% LATE DELAY; 28% MOV; 22% HOLD), whereas unbalanced spatiotopic mixed encoding was the least represented (11% VIS; 14% EARLY DELAY; 12% LATE DELAY; 11% MOV; 10% HOLD).

### Evolution of reference frames across epochs

**Figure 6:**
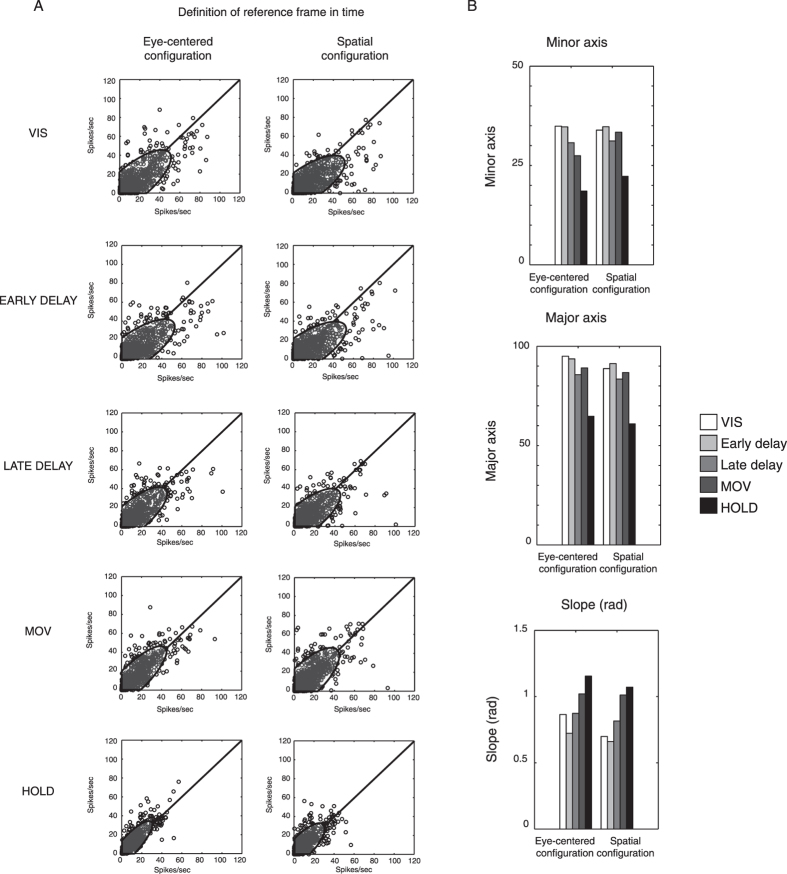
Variability analysis of the reference frames in each epoch. (**A**) left column: scatter plots of the neural activity in all epochs of pairs of movements toward targets that present the same eye/target relative position (eye-centered configuration). Right column: scatter plots of the neural activity in all epochs of pairs of movements toward targets that present the same absolute target position (spatial configuration). In each diagram, points are encircled by the ellipse that encloses the 95% of data. (**B**) Quantification of minor and major axes of ellipses and slope of longer axis in radiants.

Reference frame transformation depends on sensory availability, that changes during the execution of the trial. We measured the variability of firing rates as a parameter that indicates whether the reference frame changes across epochs^17^,^18^. If variability, expressed as data dispersion, decreased from pre-movement to movement epochs this meant that the reference frame was more stable during the motor response, and the reverse was true if the variability increased. We plotted the activity of each modulated cell in pairs of conditions during each epoch, for all the epochs: 1) when the target was constant with respect to eye position (eye-centered configuration, Fig. 6A, left column) and 2) when the target was constant in space (spatiotopic configuration, Fig. 6A, right column). The scatter plots in Fig. 6A illustrate how the firing rates of V6A cells within each frame of reference during the trial were similar. Each neuron was plotted 9 times, one for each of the 9 pairs of target positions in eye-centered and spatiotopic coordinates. To establish the statistical similarity of firing rates, we constructed the confidence ellipses (grey shadow in Fig. 6A) within which 95% of the points were located and calculated the minor and major axes, and the slope, of these ellipses (Fig. 6B). Going from pre-movement to movement phases, the ellipse shrank in both configurations, indicating that the reference frame acquired more stability (less noise) during the execution of movement. Moreover, we observed an alignment of the major axis with the diagonal that became more and more pronounced, in particular from LATE DELAY to the HOLD epoch (Fig. 6A). The length of both minor and major axes (Fig. 6B, top and middle) showed a clear decrease from EARLY DELAY to HOLD in both configurations. This suggests that the variability of the reference frame was reduced when the animal approached the target. As shown in the bottom part of Fig. 6B, the slope of the major axis was nearly coincident with that of the diagonal during the movement-related phases. Together with the reduction of variability passing from pre-movement to movement epochs, this highlights a greater constancy of neural discharge when the arm action unfolds.

### Contribution and interaction of direction and depth signals to reference frame

The reference frame of cells is strongly grounded on the interaction of depth and direction signals, both present in our task design. To examine this aspect, we measured the direction and depth resultant weights on the definition of the coordinate system, by computing a dimension index (DI, see Methods). According to this index, the weights of direction and depth were considered proportional to the lengths of x and y components of resultant vectors of gradient analysis. Positive values indicate a higher contribution of direction, negative values a higher contribution of depth. Polar plots of Figs 2B, 3B and 4B are useful for visualizing the contribution of depth and direction to vector fields. The DI of the cell of Fig. 2 showed a value of –0.87 in eye-centered configuration and −0.47 in spatiotopic configuration, suggesting a strong importance of depth dimension in the encoding process of the cell that was prominent for eye-centered configuration. The spatiotopic cell of Fig. 3, instead, showed DI values of 0.52 and 0.93 in eye-centered and spatiotopic configuration, respectively. This suggests a strong importance of direction dimension in the encoding process of the cell, more pronounced for spatiotopic configuration. Finally, the neuron of Fig. 4 shows −0.25 in eye-centered and −0.05 in spatiotopic configuration. This means that the influence of depth and direction in this cell were quite balanced.

**Figure 7:**
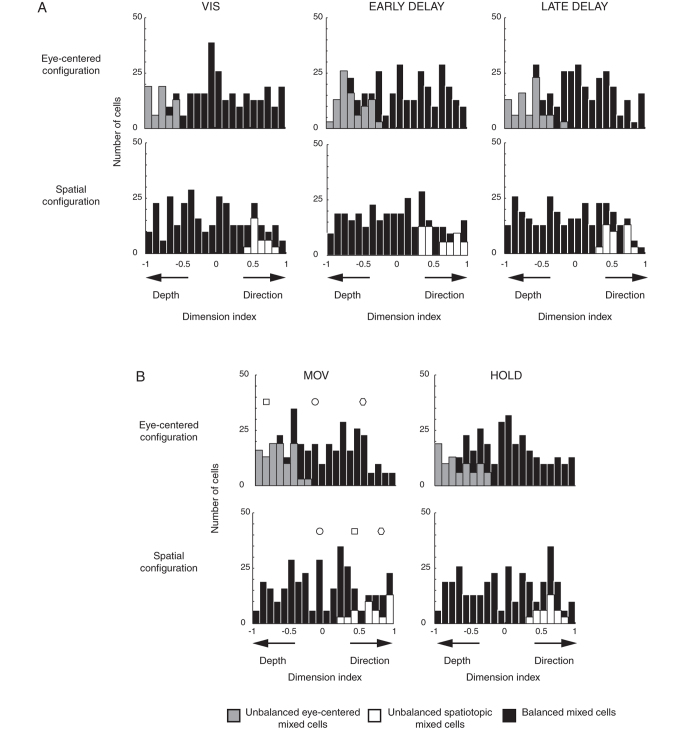
Strength of the contribution by direction and depth signals on reference frames. (**A**) Distribution of the Dimension indexes for the eye-centered configuration and the spatial configuration for pre-movement epochs. (**B**) Distribution of the Dimension indexes for the same eye-centered configuration and the spatiotopic configuration for movement epochs. Grey, white and black bins correspond to unbalanced eye-centered, unbalanced spatiotopic and balanced mixed cells, respectively. Square, circle and hexagon correspond to cells of Figs 2, 3 and 4, respectively. Balanced mixed cells show different contributions of depth and direction in all epochs of the task. Unbalanced eye-centered mixed cells show a stronger contribution of depth in all epochs, whereas unbalanced spatiotopic mixed cells show a stronger contribution of direction in the definition of reference frames.

The histograms of DIs in Fig. 7A,B illustrate that for both configurations tested in each epoch, the contribution of direction and depth to reference frame varied in each cell as the continuous distribution suggests, ranging from −1 to 1. An inspection of the allocation of unbalanced mixed cells (eye-centered and spatiotopic) within the DI distribution is particularly interesting. In fact, in each epoch, the unbalanced eye-centered mixed cells presented a preponderant role of depth in the definition of their coordinate system, as the grey columns fell in the negative part of the graph (Fig. 7A,B, eye-centered configuration). On the contrary, the unbalanced spatiotopic mixed cells were more tuned in direction, as is shown by the white columns in Fig. 7A,B (spatiotopic configuration), allocated in the positive section of the histogram distribution. Balanced mixed cells, instead, show various contributions of depth and direction, in some cases similarly and in other cases differently distributed. These results strongly indicate that the encoding of target depth is more associated with an eye-centered coordinate system, whereas target direction mainly relies on a spatiotopic coordinate system.

To determine the type of interaction between the depth and direction signals in the V6A cell we used the singular value decomposition method (SVD)^10^,^14^,^19^,^20^. The SVD is able to establish whether the pair of variables (depth and direction) of a given cell may be best described as a gain relationship where the effects of each variable on firing rate are by definition multiplicative (separable) or the two variables form part of the same function and cannot be multiplicatively separated from each other (inseparable). In this latter case, the interaction is extremely complex and the nature of the cell response is strictly based on this interaction. To define the aforementioned relationship, we employed the Constant-reach reaching task because it is the only case of the 3 tasks we used where a single variable changed (eye position with respect to reaching position). As quantified in Table 1, the number of inseparable cells in our population was much higher with respect to those presenting a separable interaction between direction and depth in both configurations analysed and in all epochs. Although each neuron presented different weights of depth and direction, their interaction showed a complex pattern in the great majority of individual cases. The example cells illustrated in Figs 2, 3 and 4 contained all inseparable forms of depth and direction interaction within the vector field considered.

## Discussion

We explored the reference frame of reaching discharges in area V6A by employing a task where eye and reaching target positions were dissociated in direction and in depth, a type of dissociation that was tested here for the first time. This allowed us to ascertain the role of vergence and version signals, as well as that of arm direction and amplitude, on neuronal discharges before, during, and after a reaching task is executed. We observed a strong presence of mixed frames of reference in V6A. Specifically, within the mixed encoding, we found that the majority of cells were balanced between eye-centered and spatiotopic representations (∼63% of neurons, averaged across epochs) and some cells displayed a mixed pattern but shifted toward an eye-centered (∼24%) and a spatiotopic representation (12%). These percentages were consistent during planning and execution of reaching movements.

A mixed encoding of target position was found in both PPC and premotor cortex^7^,^8^,^21^,^22^,^23^,^24^,^25^,^26^,^65^. Mixed encoding was also found in area V6A itself, but in tasks that did not involve depth in reaching^3^,^9^, or without eye-hand decoupling^12^. In humans, the parietal lobe shows a mixture of eye-centered and spatiotopic encoding^27^,^28^,^29^, suggesting heterogeneity of representations for visual reaching. The widespread use of the mixed representation could stem from the necessity to process different types of sensory signals without incurring in bias and variability inherent in sensorimotor transformations. In this way, the system can simultaneously represent different reference frames, reducing the noise associated with reference frame conversion^8^,^22^,^30^,^31^.

The present study shows that in a number of V6A cells (see for instance examples reported in Figs 2 and 3), the eye-centered and spatiotopic representations were not balanced, one being predominant with respect to the other. The unbalanced mixed model could be thought of as a relative code of reaching target that takes into account different variables, like eye and target position. This relative code was postulated by Pesaran *et al.*^14^ in dorsal premotor cortex (PMd), a region directly connected with V6A^32^,^33^,^34^. The unbalanced and balanced mixed patterns of discharge may also be interpreted in the light of the “decision-making theory”^35^,^36^,^37^,^38^,^39^. In this framework, there are two classes of inputs: current sensory data that reflect the actual state of the salient elements of the environment and a stored representation of environmental contingencies. Decision-making would involve the combination of actual sensory data with the subject’s best estimate of the outcome of any given action^36^. The choice of ongoing actions involves the specification of the metrics of movement, based on spatial information and on the coordinate system used by neurons. In our contest, mixed types of V6A neurons could contain an abstract representation of the two coordinate systems and then compare the current sensory state with this abstract estimation. As our task involves a complex reaching metric, a flexible model of eye-centered and spatiotopic reference frame is convenient because it allows the specification of potential reference frames weighting costs and benefits of each before arriving at the final decision. The concomitant presence of balanced and unbalanced mixed cells could be ascribed to two levels of generalization used by these neurons. The balanced mixed cells could be employed in any external condition also when the action is at early stage of planning, and the unbalanced mixed cells when the external inputs delimit the range of action^40^.

We observed that the variability of reference frames changed during the course of the task, showing a better resolution during the movement epochs with respect to the pre-movement ones. This suggests that the brain adopts the strategy to resolve and “choose” the signals to define the coordinate system to acquire the target of action when the action is actually prepared and starts. In fact, we observed that the reference frame variability was greatest during the visual epoch and the early stage of planning, and decreased progressively in the late planning and movement execution. This interpretation is in line with the “conversion-on-demand” model proposed by Henriques and coworkers^41^, according to which the reaching targets may be retained in sensory coordinates. Only the coordinates relevant to a specific action could be available to the downstream motor system^41^,^42^,^43^.

V6A shows that depth and direction contribute with different weights to the mixed reference frames. The eye-centered unbalanced mixed cells presented a higher contribution of depth signal, the spatiotopic unbalanced mixed cells showed a greater contribution of direction (Fig. 7). First of all, the depth tuning of unbalanced eye-centered mixed cells is in line with a previous study showing that reach plans encoded in the Parietal Reach Region contain a representation of target distance that is eye-centered and is modulated by vergence angle^10^. In addition, neurophysiological studies in PMd demonstrated that directional information was specified earlier in the task, during planning of movement, whereas movement distance displayed its effect mostly during movement execution^44^,^45^. A similar discharge pattern was recently observed in the medial PPC area V6A, with an increase of depth-modulated neurons and depth sensitivity as the task progressed^46^. Typically, the information about target location is encoded first in the eye-centered frame of reference and then into a coordinate system referring to the body, hand or space^1^. This transformation takes place serially in the PPC, that is, caudal PPC areas encode reach targets in the eye-centered reference frame, whereas rostral ones use spatiotopic coordinates^2^,^3^,^4^,^5^. Area V6A is a caudal area of the PPC and represents an early node of visuomotor transformation. Combining all these data, the findings that the unbalanced spatiotopic mixed encoding is more frequent in direction and the unbalanced eye-centered mixed encoding is more frequent in depth could be explained by the timing of coordinate transformation. In fact, given that depth is processed later with respect to direction, it might be the case that some neurons, in direction, have already transformed eye-centered to spatiotopic coordinates, whereas in depth this transformation has not yet occurred.

Behavioural evidence supports the view that depth and direction signals were processed independently^1^,^47^,^48^,^49^,^50^,^51^. We recently reported that many V6A cells are modulated by both vergence/depth and version/direction signals^46^,^52^, and therefore are able to encode the 3D spatial coordinates of foveated reaching targets. Here, we found that depth and direction signals modulate the activity of neurons also in decoupled reaching movement. In a few cases, the relationship between depth and direction was explained by gain modulation; in most cases, the two variables show complex interactions, as demonstrated by the high percentage of inseparable cells (Table 1). This interaction is useful to maintain spatial constancy in eye-centered coordinates^53^,^54^,^55^,^56^ and their close correlation suggests a joint processing within the same cell, adding new data to previous demonstrations that in V6A there are single neurons jointly encoding target depth and direction^46^.

The present results show that when we perform reaching in a 3D space, V6A neurons use at least a double reference frame. This supports the view suggested by many authors in studies in humans and monkeys^21^,^27^,^29^ that the reference frame options for the execution of an accurate 3D reach are expressed in terms of mixed and flexible models of eye-centered and spatiotopic coordinate systems within the PPC.

## Materials and Methods

### General procedures

Two male macaque monkeys (*Macaca fascicularis*) participated in this study. All surgical and animal care procedures were conducted in accordance with the European Directive 2010/63/EU. All the experimental protocols were approved by the Bioethics Committee of the University of Bologna. During training and recording sessions, particular care was taken to avoid any behavioral and clinical sign of pain or distress.

A head-restraint system and a recording chamber were surgically implanted under general anesthesia (sodium thiopental, 8 mg/kg*h, i.v.) following the criteria reported in Galletti *et al.*^57^. A full program of postoperative analgesia (ketorolac tromethamine, 1 mg/kg i.m. immediately after surgery, and 1.6 mg/kg i.m. on the following days) and antibiotic care (Ritardomicin, benzatine benzylpenicillin + dihydrostreptomycin, 1–1.4 ml/10 kg every 5–6 days) followed the surgery.

Extracellular recording techniques to reconstruct microelectrode penetrations were similar to those described in other reports^58^. Single cell activity was extracellularly recorded from the anterior bank of the parieto-occipital sulcus with the aim of studying V6A neurons. Area V6A was recognized during recordings on functional grounds, following the criteria described in Galletti *et al.*^13^, and later confirmed with the cytoarchitectonic criteria described by Luppino *et al.*^59^. We performed multiple electrode penetrations using a 5-channel multielectrode recording system (Thomas Recording). The action potentials were amplified (gain 10000) and filtered (band pass between 0.5 and 5 kHz). A waveform discriminator allowed the isolation of action potentials in each channel (Multi Spike Detector; Alpha Omega Engineering) and they were sampled at 100 kHz.

### Behavioral paradigm

Electrophysiological signals were collected while monkeys were performing a reaching task in darkness using the hand contralateral to the recording hemisphere and with the head restrained. As shown in Fig. 1A, reaching movements started from a button placed near the monkey’s chest (4 cm from it), on the mid-sagittal line and outside the animal’s field of view. The animal reached for 9 Light Emitting Diodes (LEDs) positioned at eye level, at three different distances and in three different directions (Fig. 1A,B). Three LED targets were placed at three isovergence angles: the nearest targets were located at 10 cm from the eyes (17.1° of vergence) and the LEDs located at intermediate and far positions were at a depth of 15 cm (11.4°) and 25 cm (6.9°), respectively. At each isovergence angle, LEDs were positioned in three directions: one central, along the sagittal midline and 2 lateral, at iso-version angles of −15° and +15°. Target positions were selected in order to be all within the peripersonal space. The time sequence of the task is shown in Fig. 1C. A trial began when the monkey pressed the button (HB press). The animal was free to look around, and was not required to immediately perform any eye or arm movement. After 1000 ms, the central LED lit up (LED on), which signaled the monkey to gaze at it and to keep the button pressed down while awaiting the instructional cue. After a delay of 1000 ms, a yellow LED in one of the 9 target positions was illuminated for 150 ms, indicating the target for the reaching movement. The monkey then had to wait an additional 1000–1500 ms for a change in color of the fixation LED (green to red) without performing any eye or arm movement. The color change of the fixation target was the go-signal for the monkey to release the home button and, while maintaining fixation, perform an arm movement toward the reaching target and press it. The animal held its hand on the reaching target until the fixation LED switched off (after 800–1200 ms). The offset of the fixation LED informed the monkey to release the reaching target, and to press the home button again to be rewarded and start another trial.

The task consisted in 27 trials distributed in three blocks and randomly interleaved within each block. In the first block, the reaching movement was executed maintaining gaze fixation on the central, straight-ahead position (Fig. 1B, Constant-gaze reaching task). Keeping the fixation point constant allowed constant vergence and version eye signals and precluded cell responses resulting from the eye vergence and version signals, known to affect V6A neural discharges^52^,^60^.

The second block, called Constant-reach reaching task, consisted in a task where the target of reaching was constantly located in a central position (Fig. 1B) whereas the fixation point was located in one of the 9 peripheral positions. Keeping the reaching target constant allowed a constant distance and direction of reaching movements, and thus precluded cell responses resulting from the distance and direction of arm movement, known to affect V6A neural discharges^46^. This condition allowed us to manipulate the eye/target relative coordinates of the target, while keeping the spatial position of reaching target constant.

The last block was identified as Foveal reaching task (Fig. 1B). Here, the fixation target was always coincident with the reaching target. Since reaching movements are always directed toward foveated targets, the eye/target relative coordinates of the reaching target remained constant throughout the task, while both eye version/vergence and arm direction/depth were continuously changing. The time sequence, LED position, task control and other parameters of the 3 task blocks were exactly the same as those described above.

An electronic window (4° × 4°) forced the monkeys to fixate on the LED from LED onset (before the go signal) until LED offset (that cued the return reach). If fixation was broken during this interval, trials were interrupted on-line and discarded. Correct performance of reaching movements was detected via monopolar microswitches (RS Components, UK) mounted under the home button and the reaching targets. Button presses/releases were recorded with 1 ms resolution. The presentation of stimuli and the animal performance were monitored using custom software written in Labview (National Instruments), as described previously^61^. Eye position signals were sampled with 2 cameras (1 for each eye) of an infrared oculometer system (ISCAN) at 100 Hz. The background light was switched on briefly between blocks to avoid dark adaptation.

At the start of each session, monkeys were required to perform a calibration task where they fixated 10 LEDs mounted on a frontal panel at a distance of 15 cm from the eyes. For each eye, signals to be used for calibration were extracted during fixation of 5 LEDs, 1 central aligned with the eye’s straight ahead position and 4 peripheral placed at an angle of ±15° (distance: 4 cm) both in the horizontal and vertical axes. From the 2 individual calibrated eye position signals, we derived the mean of the 2 eyes (the conjugate or version signal), and the difference between the 2 eyes (the disconjugate or vergence signal) using the equations: version = (R + L)/2 and vergence = R − L, where R and L was the position of the right and left eye, respectively.

### Data analysis

Only single units with a minimum of seven trials per condition were included in the dataset^62^. Data analysis was performed trial-by-trial. For each trial we screened for a correlation between neural discharge, eye-position and/or target position. As shown in the bottom part of Fig. 1C, the functional epochs defined in this study were:

- VIS: visual period from 40 ms after yellow cue onset (Target on) to 150 ms after yellow cue offset (Target off);
- EARLY delay: delay period between 300 ms and 650 ms after the yellow cue onset (Target on);
- LATE delay: period that corresponded to the last 500 ms before the go signal (GO);
- MOV: epoch that started 200 ms before home button release (M) and ended at the end of the reaching movement (H);
- HOLD: period from the end of forward reach (H) to the offset of fixation target (Red off).

As a reference period, we calculated an epoch from the pressing of the home button to the illumination of the green LED (epoch FREE).

As we tested the cells in different blocks, our first concern was to check for neuronal recording stability across the blocks. We used the interspike interval analysis to ensure that changes in neuronal modulations were not caused by simply losing isolation of the neuron. Additionally, to ensure that the recording situation did not change across the 3 different blocks, we compared the cell activity during epoch FREE in the central position (that was repeated in each task configuration) of the three blocks by applying Student’s t-test (P < 0.05). Only cells conforming to all the above criteria were included in the analysis.

One-way ANOVA (factor: target position; dependent variable: activity during VIS, EARLY DELAY, LATE DELAY, MOV, HOLD) was used to compare neural activity within epochs and blocks. Cells showing a significant effect of target position in at least one of the three blocks for at least one epoch of the task were inserted in the analyses.

Gradient analysis^4^,^14^,^15^,^16^ was used to determine which variable within a pair of eye/target configurations (eye-centered and spatiotopic configurations) exerted the most influence on the firing rate of a cell, or whether the configurations had equivalent influence. The gradient of the response matrix was estimated with the Matlab gradient function (Matworks^®^). The x and y component of the 2 vector fields corresponding to the pair of the eye-centered and spatial configurations, respectively, were summed together in order to obtain two resultant vectors (eye-centered and spatial resultant vector) defined by the length and the angle with respect to the horizontal axis (see Fig. 2B as example). The reference frame of cells was ascertained if the resultant length was greater or smaller than the resultant length calculated after randomization of the matrix elements (randomization test, 10000 iterations) and the resultant angles were used to evaluate the overall spatial distribution of cell firing rates in the different eye/target configuration tested (i.e. Fig. 2B). The randomization allowed us to extract confidence intervals that included 95% of values for each pair of configurations analysed (first pair: eye-centered configurations; second pair: spatial configurations). As the eye-centered representation tends to have opposite directions of vector fields in Constant-gaze and Constant-reach configurations, we defined cells as unbalanced eye-centered mixed cells when the resultant vector was smaller than the lower confidence interval (CI) for this pair of configurations (Fig. 2C), and as unbalanced spatiotopic mixed cells when the resultant vector was larger than the upper CI extracted from the vector fields of Constant-gaze and Foveal reach (Fig. 3C), as the vector fields in these configurations present the same direction. We defined those cells that showed resultant vectors not responding to previous criteria as balanced mixed cells (Fig. 4C).

To study the evolution of the reference frame during the execution of the trial, we compared the mean firing rates of single conditions: 1) when targets had the same location relative to the eye and 2) when targets had the same location in the peripersonal space of the monkey. At the population level, the similarity of paired firing rates was evaluated by calculating confidence ellipses for 2D normally distributed data, as usually done in psychophysical studies^63^ (see Fig. 6A). The shape of the confidence ellipses was determined by computing the eigenvectors and eigenvalues. Eigenvectors represent the direction in which the data varies the most and the eigenvalues correspond to the spread of the data in the direction of the eigenvectors. The 95% confidence ellipse was calculated from the length of the major and minor axes defined by standard deviations σ_x_ and σ_y_ of the data. The orientation of the ellipse was calculated from the angle of the largest eigenvector toward the x-axis (Fig. 6B). We extracted the slope of the ellipse in radiants. The lengths of the major and minor axes and their slope were correlated to the amount of variability that describes the definition level of reference frames.

A dimension index (DI) was carried out to see the contribution of depth and direction signals in the recruitment of reference frame used by V6A cells. DI quantified the weight of depth and direction dimensions in the two pairs of eye and spatial configuration tested (see Fig. 7) and is calculated as follows:


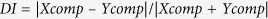


where Xcomp and Ycomp are x and y components of resultant vectors calculated for each pair of task configurations representing direction and depth signals, respectively (or put in eye terms, version and vergence angles). The vector components were normalized between −1 and 1, where values near −1 indicate neural responses mostly influenced by depth and values near 1 mostly influenced by direction.

The type of interaction between depth and direction signals was given by the application of the singular value decomposition analysis or SVD^10^,^12^,^14^,^19^,^20^. SVD defines whether pairs of variables are related by a multiplicative or gain relationship (separable) or interact in a complex way (inseparable). A 3 × 3 matrix representing the Constant-reach combination was constructed from the mean activity across eye and target conditions. This matrix was subsequently reconstructed to calculate the diagonal matrix S that contained the singular values. Neural responses were classified as separable if the first singular value was significantly larger (P < 0.05) than the first singular value calculated when conditions were randomized by permutating the rows and columns of the initial matrix^10^,^12^,^14^.

## Additional Information

**How to cite this article**: Bosco, A. *et al.* Reference frames for reaching when decoupling eye and target position in depth and direction. *Sci. Rep.* **6**, 21646; doi: 10.1038/srep21646 (2016).

## Figures and Tables

**Table 1:** Incidence of separable and inseparable cells in Constant-reach reaching task.

	Constant-reach reaching task	
	Separable	Inseparable
VIS	3/99 (3%)	96/99 (97%)
Early delay	4/105 (3%)	101/105 (97%)
Late delay	4/102 (4%)	98/102 (96%)
MOV	11/106 (10%)	95/106 (90%)
HOLD	6/104 (6%)	98/104 (94%)

## References

[b1] Flanders M., Tillery, S.I. H. & Soechting & J. F. S. Early stages in a sensorimotor transformation. Behav Brain Sci 15, 309–362 (1992).

[b2] Batista A. P., Buneo C. A., Snyder L. H. & Andersen R. A. Reach plans in eye-centered coordinates. Science 285, 257–260 (1999). 10398603 10.1126/science.285.5425.257

[b3] Marzocchi N., Breveglieri R., Galletti C. & Fattori P. Reaching activity in parietal area V6A of macaque: eye influence on arm activity or retinocentric coding of reaching movements? Eur J Neurosci 27, 775–789, 10.1111/j.1460-9568.2008.06021.x (2008). 18279330 PMC2268963

[b4] Buneo C. A., Jarvis M. R., Batista A. P. & Andersen R. A. Direct visuomotor transformations for reaching. Nature 416, 632–636, 10.1038/416632a (2002). 11948351

[b5] Buneo C. A., Batista A. P., Jarvis M. R. & Andersen R. A. Time-invariant reference frames for parietal reach activity. Exp Brain Res 188, 77–89, 10.1007/s00221-008-1340-x (2008). 18368398

[b6] Chang S. W., Papadimitriou C. & Snyder L. H. Using a compound gain field to compute a reach plan. Neuron 64, 744–755, 10.1016/j.neuron.2009.11.005 (2009). 20005829 PMC2811884

[b7] Mullette-Gillman O. A., Cohen Y. E. & Groh J. M. Motor-related signals in the intraparietal cortex encode locations in a hybrid, rather than eye-centered reference frame. Cereb Cortex 19, 1761–1775, 10.1093/cercor/bhn207 (2009). 19068491 PMC2705694

[b8] McGuire L. M. & Sabes P. N. Heterogeneous representations in the superior parietal lobule are common across reaches to visual and proprioceptive targets. J Neurosci 31, 6661–6673, 10.1523/JNEUROSCI.2921-10.2011 (2011). 21543595 PMC3100795

[b9] Bosco A., Breveglieri R., Reser D., Galletti C. & Fattori P. Multiple Representation of Reaching Space in the Medial Posterior Parietal Area V6A. Cereb Cortex 25, 1654–1667, 10.1093/cercor/bht420 (2015). 24421176

[b10] Bhattacharyya R., Musallam S. & Andersen R. A. Parietal reach region encodes reach depth using retinal disparity and vergence angle signals. J Neurophysiol 102, 805–816, 10.1152/jn.90359.2008 (2009). 19439678 PMC2724352

[b11] Ferraina S. *et al.* Reaching in depth: hand position dominates over binocular eye position in the rostral superior parietal lobule. J Neurosci 29, 11461–11470, 10.1523/JNEUROSCI.1305-09.2009 (2009). 19759295 PMC6665750

[b12] Hadjidimitrakis K., Bertozzi F., Breveglieri R., Fattori P. & Galletti C. Body-Centered, Mixed, but not Hand-Centered Coding of Visual Targets in the Medial Posterior Parietal Cortex During Reaches in 3D Space. Cereb Cortex 24, 3209–3220, 10.1093/cercor/bht181 (2014). 23853212

[b13] Galletti C., Fattori P., Kutz D. F. & Gamberini M. Brain location and visual topography of cortical area V6A in the macaque monkey. Eur J Neurosci 11, 575–582 (1999). 10051757 10.1046/j.1460-9568.1999.00467.x

[b14] Pesaran B., Nelson M. J. & Andersen R. A. Dorsal premotor neurons encode the relative position of the hand, eye, and goal during reach planning. Neuron 51, 125–134, 10.1016/j.neuron.2006.05.025 (2006). 16815337 PMC3066049

[b15] Pesaran B., Nelson M. J. & Andersen R. A. A relative position code for saccades in dorsal premotor cortex. J Neurosci 30, 6527–6537, 10.1523/JNEUROSCI.1625-09.2010 (2010). 20463216 PMC2887302

[b16] Bremner L. R. & Andersen R. A. Temporal analysis of reference frames in parietal cortex area 5d during reach planning. J Neurosci 34, 5273–5284, 10.1523/JNEUROSCI.2068-13.2014 (2014). 24719105 PMC3983803

[b17] Sober S. J. & Sabes P. N. Flexible strategies for sensory integration during motor planning. Nat Neurosci 8, 490–497, 10.1038/nn1427 (2005). 15793578 PMC2538489

[b18] Schlicht E. J. & Schrater P. R. Effects of visual uncertainty on grasping movements. Exp Brain Res 182, 47–57, 10.1007/s00221-007-0970-8 (2007). 17503025

[b19] Peña J. L. & Konishi M. Auditory spatial receptive fields created by multiplication. Science 292, 249–252, 10.1126/science.1059201 (2001). 11303092

[b20] Blohm G. Simulating the cortical 3D visuomotor transformation of reach depth. PLoS One 7, e41241, 10.1371/journal.pone.0041241 (2012). 22815979 PMC3397995

[b21] Chang S. W. & Snyder L. H. Idiosyncratic and systematic aspects of spatial representations in the macaque parietal cortex. Proc Natl Acad Sci USA 107, 7951–7956, 10.1073/pnas.0913209107 (2010). 20375282 PMC2867917

[b22] Avillac M., Denève S., Olivier E., Pouget A. & Duhamel J. R. Reference frames for representing visual and tactile locations in parietal cortex. Nat Neurosci 8, 941–949, 10.1038/nn1480 (2005). 15951810

[b23] Battaglia-Mayer A., Caminiti R., Lacquaniti F. & Zago M. Multiple levels of representation of reaching in the parieto-frontal network. Cereb Cortex 13, 1009–1022 (2003). 12967918 10.1093/cercor/13.10.1009

[b24] Cohen Y. E. & Andersen R. A. A common reference frame for movement plans in the posterior parietal cortex. Nat Rev Neurosci 3, 553–562, 10.1038/nrn873 (2002). 12094211

[b25] Mullette-Gillman O. A., Cohen Y. E. & Groh J. M. Eye-centered, head-centered, and complex coding of visual and auditory targets in the intraparietal sulcus. J Neurophysiol 94, 2331–2352, 10.1152/jn.00021.2005 (2005). 15843485

[b26] Stricanne B., Andersen R. A. & Mazzoni P. Eye-centered, head-centered, and intermediate coding of remembered sound locations in area LIP. J Neurophysiol 76, 2071–2076 (1996). 8890315 10.1152/jn.1996.76.3.2071

[b27] Bernier P. M. & Grafton S. T. Human posterior parietal cortex flexibly determines reference frames for reaching based on sensory context. Neuron 68, 776–788, 10.1016/j.neuron.2010.11.002 (2010). 21092865

[b28] Beurze S. M., Toni I., Pisella L. & Medendorp W. P. Reference frames for reach planning in human parietofrontal cortex. J Neurophysiol 104, 1736–1745, 10.1152/jn.01044.2009 (2010). 20660416

[b29] Buchholz V. N., Jensen O. & Medendorp W. P. Parietal oscillations code nonvisual reach targets relative to gaze and body. J Neurosci 33, 3492–3499, 10.1523/JNEUROSCI.3208-12.2013 (2013). 23426676 PMC6619549

[b30] Deneve S., Latham P. E. & Pouget A. Efficient computation and cue integration with noisy population codes. Nat Neurosci 4, 826–831, 10.1038/90541 (2001). 11477429

[b31] McGuire L. M. & Sabes P. N. Sensory transformations and the use of multiple reference frames for reach planning. Nat Neurosci 12, 1056–1061, 10.1038/nn.2357 (2009). 19597495 PMC2749235

[b32] Gamberini M. *et al.* Cortical connections of the visuomotor parietooccipital area V6Ad of the macaque monkey. J Comp Neurol 513, 622–642, 10.1002/cne.21980 (2009). 19235224

[b33] Shipp S., Blanton M. & Zeki S. A visuo-somatomotor pathway through superior parietal cortex in the macaque monkey: cortical connections of areas V6 and V6A. Eur J Neurosci 10, 3171–3193 (1998). 9786211 10.1046/j.1460-9568.1998.00327.x

[b34] Matelli M., Govoni P., Galletti C., Kutz D. F. & Luppino G. Superior area 6 afferents from the superior parietal lobule in the macaque monkey. J Comp Neurol 402, 327–352 (1998). 9853903

[b35] Carpenter R. H. & Williams M. L. Neural computation of log likelihood in control of saccadic eye movements. Nature 377, 59–62, 10.1038/377059a0 (1995). 7659161

[b36] Platt M. L. & Glimcher P. W. Neural correlates of decision variables in parietal cortex. Nature 400, 233–238, 10.1038/22268 (1999). 10421364

[b37] Mazurek M. E., Roitman J. D., Ditterich J. & Shadlen M. N. A role for neural integrators in perceptual decision making. Cereb Cortex 13, 1257–1269 (2003). 14576217 10.1093/cercor/bhg097

[b38] Smith P. L. & Ratcliff R. Psychology and neurobiology of simple decisions. Trends Neurosci 27, 161–168, 10.1016/j.tins.2004.01.006 (2004). 15036882

[b39] Cisek P. & Kalaska J. F. Neural correlates of reaching decisions in dorsal premotor cortex: specification of multiple direction choices and final selection of action. Neuron 45, 801–814, 10.1016/j.neuron.2005.01.027 (2005). 15748854

[b40] Gibson J.J. The ecological approach to visual perception. Boston:Houghton Mifflin (1979).

[b41] Henriques D. Y., Klier E. M., Smith M. A., Lowy D. & Crawford J. D. Gaze-centered remapping of remembered visual space in an open-loop pointing task. J Neurosci 18, 1583–1594 (1998). 9454863 10.1523/JNEUROSCI.18-04-01583.1998 PMC6792733

[b42] Klier E. M., Wang H. & Crawford J. D. The superior colliculus encodes gaze commands in retinal coordinates. Nat Neurosci 4, 627–632, 10.1038/88450 (2001). 11369944

[b43] Lee J. & Groh J. M. Auditory signals evolve from hybrid- to eye-centered coordinates in the primate superior colliculus. J Neurophysiol 108, 227–242, 10.1152/jn.00706.2011 (2012). 22514295 PMC3434609

[b44] Fu Q. G., Suarez J. I. & Ebner T. J. Neuronal specification of direction and distance during reaching movements in the superior precentral premotor area and primary motor cortex of monkeys. J Neurophysiol 70, 2097–2116 (1993). 8294972 10.1152/jn.1993.70.5.2097

[b45] Messier J. & Kalaska J. F. Covariation of primate dorsal premotor cell activity with direction and amplitude during a memorized-delay reaching task. J Neurophysiol 84, 152–165 (2000). 10899193 10.1152/jn.2000.84.1.152

[b46] Hadjidimitrakis K. *et al.* Common neural substrate for processing depth and direction signals for reaching in the monkey medial posterior parietal cortex. Cereb Cortex 24, 1645–1657, 10.1093/cercor/bht021 (2014). 23382514

[b47] Gordon J., Ghilardi M. F. & Ghez C. Accuracy of planarreaching movements. I. Independence of direction and extent variability. Exp Brain Res 99, 97–111 (1994). 7925800 10.1007/BF00241415

[b48] Sainburg R. L., Lateiner J. E., Latash M. L. & Bagesteiro L. B. Effects of altering initial position on movement direction and extent. J Neurophysiol 89, 401–415 10.1152/jn.00243.2002 (2003). 12522189 PMC10709819

[b49] Vindras P., Desmurget M. & Viviani P. Error parsing in visuomotor pointing reveals independent processing of amplitude and direction. J Neurophysiol 94, 1212–1224, 10.1152/jn.01295.2004 (2005). 15857965

[b50] Bagesteiro L. B., Sarlegna F. R. & Sainburg R. L. Differential influence of vision and proprioception on control of movement distance. Exp Brain Res 171, 358–370, 10.1007/s00221-005-0272-y (2006). 16307242 PMC10710692

[b51] Van Pelt S. & Medendorp W. P. Updating target distance across eye movements in depth. J Neurophysiol 99, 2281–2290, 10.1152/jn.01281.2007 (2008). 18353912

[b52] Breveglieri R. *et al.* Eye position encoding in three-dimensional space: integration of version and vergence signals in the medial posterior parietal cortex. J Neurosci 32, 159–169, 10.1523/JNEUROSCI.4028-11.2012 (2012). 22219279 PMC6621321

[b53] Li N. & Angelaki D. E. Updating visual space during motion in depth. Neuron 48, 149–158, 10.1016/j.neuron.2005.08.021 (2005). 16202715

[b54] Medendorp W. P. & Crawford J. D. Visuospatial updating of reaching targets in near and far space. Neuroreport 13, 633–636 (2002). 11973460 10.1097/00001756-200204160-00019

[b55] Medendorp W. P., Goltz H. C., Vilis T. & Crawford J. D. Gaze-centered updating of visual space in human parietal cortex. J Neurosci 23, 6209–6214 (2003). 12867504 10.1523/JNEUROSCI.23-15-06209.2003 PMC6740538

[b56] Van Pelt S. & Medendorp W. P. Gaze-centered updating of remembered visual space during active whole-body translations. J Neurophysiol 97, 1209–1220, 10.1152/jn.00882.2006 (2007). 17135474

[b57] Galletti C., Battaglini P. P. & Fattori P. Eye position influence on the parieto-occipital area PO (V6) of the macaque monkey. Eur J Neurosci 7, 2486–2501 (1995). 8845954 10.1111/j.1460-9568.1995.tb01047.x

[b58] Gamberini M., Galletti C., Bosco A., Breveglieri R. & Fattori P. Is the medial posterior parietal area V6A a single functional area? J Neurosci 31, 5145–5157, 10.1523/JNEUROSCI.5489-10.2011 (2011). 21451050 PMC6622963

[b59] Luppino G., Ben Hamed S., Gamberini M., Matelli M. & Galletti C. Occipital (V6) and parietal (V6A) areas in the anterior wall of the parieto-occipital sulcus of the macaque: a cytoarchitectonic study. Eur J Neurosci 21, 3056–3076, 10.1111/j.1460-9568.2005.04149.x (2005). 15978016

[b60] Breveglieri R., Galletti C., Bosco A., Gamberini M. & Fattori P. Object Affordance Modulates Visual Responses in the Macaque Medial Posterior Parietal Cortex. J Cogn Neurosci, 1–9, 10.1162/jocn_a_00793 (2015). 25647337

[b61] Kutz D. F., Marzocchi N., Fattori P., Cavalcanti S. & Galletti C. Real-time supervisor system based on trinary logic to control experiments with behaving animals and humans. J Neurophysiol 93, 3674–3686, 10.1152/jn.01292.2004 (2005). 15703220

[b62] Kutz D. F., Fattori P., Gamberini M., Breveglieri R. & Galletti C. Early- and late-responding cells to saccadic eye movements in the cortical area V6A of macaque monkey. Exp Brain Res 149, 83–95, 10.1007/s00221-002-1337-9 (2003). 12592506

[b63] Davare M., Zénon A., Desmurget M. & Olivier E. Dissociable contribution of the parietal and frontal cortex to coding movement direction and amplitude. Front Hum Neurosci 9, 241, 10.3389/fnhum.2015.00241 (2015). 25999837 PMC4422032

[b64] Buneo C. A. & Andersen R. A. Integration of target and hand position signals in the posterior parietal cortex: effects of workspace and hand vision. J Neurophysiol 108, 187–199, 10.1152/jn.00137.2011 (2012). 22457457 PMC3434607

[b65] Batista A. P. *et al.* Reference frames for reach planning in macaque dorsal premotor cortex. J Neurophysiol 98, 966–983, 10.1152/jn.00421.2006 (2007). 17581846

